# Genomic Epidemiology and Evolution of Fowl Adenovirus 1

**DOI:** 10.3390/ani13182819

**Published:** 2023-09-05

**Authors:** Szilvia Jakab, Krisztina Bali, Zalán Homonnay, Eszter Kaszab, Katalin Ihász, Enikő Fehér, Tamás Mató, István Kiss, Vilmos Palya, Krisztián Bányai

**Affiliations:** 1 Veterinary Medical Research Institute, Hungária krt. 21, H-1143 Budapest, Hungary; jakab.szilvia@vmri.hu (S.J.); bali.krisztina@vmri.hu (K.B.); kaszab.eszter@vmri.hu (E.K.); ihasz.katalin@vmri.hu (K.I.); feher.eniko@vmri.hu (E.F.); 2 National Laboratory for Infectious Animal Diseases, Antimicrobial Resistance, Veterinary Public Health and Food Chain Safety, Hungária krt. 21, H-1143 Budapest, Hungary; 3 Ceva-Phylaxia Ltd., Szállás u. 5, H-1107 Budapest, Hungary; zalan.homonnay@ceva.com (Z.H.); tamas.mato@ceva.com (T.M.); istvan.kiss@ceva.com (I.K.); vilmos.palya@ceva.com (V.P.); 4 Department of Pharmacology and Toxicology, University of Veterinary Medicine, István utca 2, H-1078 Budapest, Hungary

**Keywords:** *Fowl adenovirus A*, gizzard erosion, genotyping, phylogenetic analysis, recombination

## Abstract

**Simple Summary:**

Gizzard erosion is an important pathology in chickens. Aviadenoviruses, in particular, fowl adenovirus 1, have been associated with the etiology of gizzard erosion. Clinical and pathology data from the field and from infection experiments suggested that both virulent and avirulent strains are circulating, although the genetic differences between these two FAdV-1 pathotypes have not been clarified. In this study, we analyzed a total of 40 FAdV-1 genomes, including 32 newly determined genome sequences, and demonstrated that a set of viral genes differ phylogenetically between the two pathotypes. These findings may help select suitable naturally attenuated FAdV-1 strains that can be used as an effective live vaccine acting directly on the mucosal surface of the gizzard.

**Abstract:**

Fowl adenovirus 1 (FAdV-1) is the main cause of gizzard erosion in chickens. Whole genome sequencing and sequence analyses of 32 FAdV-1 strains from a global collection provided evidence that multiple recombination events have occurred along the entire genome. In gene-wise phylogenies, only the adenoviral pol gene formed a tree topology that corresponded to whole genome-based phylogeny. Virus genetic features that were clearly connected to gizzard erosion were not identified in our analyses. However, some genome variants tended to be more frequently identified from birds with gizzard erosion and strains isolated from healthy birds or birds with non-specific pathologies tended to form common clusters in multiple gene phylogenies. Our data show that the genetic diversity is greater, and the evolutionary mechanisms are more complex within FAdV-1 than previously thought. The implications of these findings for viral pathogenesis and epidemiology await further investigation.

## 1. Introduction

Adenoviruses are middle-sized, non-enveloped, double-stranded DNA viruses, which infect all major classes of vertebrates [1]. Fowl adenoviruses (FAdVs) may cause a variety of diseases in chickens. The most commonly reported clinicopathological manifestations of FAdV infections are gizzard erosion (GE), inclusion body hepatitis, hydropericardium hepatitis syndrome in chicken, and bronchitis in quail [2].

FAdVs belong to the aviadenovirus genus. FAdVs are classified into five species (designated as fowl aviadenovirus A to fowl aviadenovirus E) and are further classified into 12 serotypes (designated 1 to 11 where serotypes 8a and 8b are also distinguished) by cross-neutralization assays [3]. Unlike FAdV-C to FAdV-E, which are multi-serotype FAdV species, FAdV-A and FAdV-B are single-serotype FAdVs. The only known serotype within FADV-A has been designated serotype 1 (FAdV-1).

The genome of reference FAdV-A strain, CELO (derived from the term ‘chicken embryo lethal orphan’), is 43,804 bp in length and encodes at least 39 genes [1,4]. Among FAdV species and serotypes, FAdV-A (FAdV-1) has been considered the genetically least variable, as all strains characterized so far seem to share ≥99% nucleotide similarities [5]. The genetic diversification of FAdV-1 strains is primarily based on the accumulation of point mutations and indel mutations, the latter aggregating chiefly in the tandem-repeat regions [6,7]. Mutations leading to amino acid changes in the coding regions are scarce in all identified FAdV-1 genomes [6,8,9,10].

To gain further insight into the molecular epidemiology and the genomic evolution of FAdV-1, we report here the genome of 32 FAdV-1 isolates originating from an international collection. We also evaluated the available data to uncover the genomic regions potentially associated with viral pathology.

## 2. Materials and Methods

### 2.1. Virus Strains

The FAdV-1 strains were isolated from pathological samples as part of routine diagnostic procedures at CEVA-Phylaxia and identified by seminested PCR [11]. The chicken hepatocellular carcinoma cell line LMH was used to isolate virus from homogenates of organs (including the intestine, cecal tonsil, liver, gizzard, trachea, lung, pancreas, joint, or bursa). All FAdV-1 isolates were stored at −70 °C until DNA extraction and genome sequencing. The isolates originated from 15 countries and four continents encompassing 12 years [11] (see Table S1).

### 2.2. Sequencing

Next-generation sequencing was used to obtain the consensus genomic sequences. The viral nucleic acid was extracted directly from 200 µL cell culture supernatants using the innuPREP Virus DNA/RNA Kit (Analytik Jena, Jena, Germany) according to the manufacturer’s instructions. The reversible terminator sequencing method was used to determine whole genome sequences as described elsewhere in detail [12,13]. DNA libraries were sequenced on an Illumina^®^ NextSeq 500 sequencer (Illumina, San Diego, CA, USA). Sanger sequencing was carried out to confirm the structure of the tandem-repeat region bridging ORF19 and ORF8. The primer sequences used in PCR amplification and direct sequencing were as follows: 5′-CTCACAGTCTATTGCAGACTGC-3′ and 5′-CACTAGCCTTAGAGAAATGGTG-3′. Dye-labeled fragments were run on ABI 3500 equipment (Delta-Bio Ltd., Szeged, Hungary). Genome assemblies were carried out by combining the sequence files from NGS runs and capillary sequencing runs within the Geneious Prime^®^ 2022.2.2 software (Biomatters Ltd., Auckland, New Zealand). The obtained sequences were deposited in GenBank under the accession numbers OP985603 to OP985634.

### 2.3. Sequence Analysis

Multiple alignments of the individual genes as well as the complete genomes were prepared by using MAFFT aligner and visualized in Geneious. Gene-wise and whole-genome-based phylogenies were performed with the maximum-likelihood method using the Hasegawa-Kishino-Yano model or the Tamura 3-parameter model in MEGA 11 (version 11.0.13.) [14] and IQ-TREE web server [15]. Additionally, the whole genomes were used to test whether recombination occurred among different FAdV-A strains using the available algorithms implemented in the Recombination Detection Program 5 (RDP5, version 5.5) [16]. Default settings were used for each algorithm. A recombination event was accepted when detected by 7 distinct methods (RDP, GENECONV, BootScan, MaxChi, Chimaera, SiScan, 3Seq) implemented in the program, with a *p*-value less than 5 × 10^−4^.

## 3. Results

### 3.1. Features of FAdV-1 Genomes

From our strain collection that contained around 40 FAdV-1 isolates [11], 32 were available for whole genome sequencing. Another eight FAdV-1 genome sequences from GenBank were added to downstream analyses. The length of genomes showed little variation (genome sizes were approximately 43.6 to 43.8 kbp). We note here that no efforts were made to determine the true 5′ and 3′ ends of the genomic DNA. The sequence coverage fell consistently above 99.9%, whereas the average sequencing depth varied between 25× and 3712× (average, 1541×; median 1449×; × stands for multiply). We observed some variations in the length of 11 ORFs when comparing the genome of study strains with the reference strain, CELO (see Table S2).

The true sequence of the tandem-repeat region bridging ORF19 and ORF8 required confirmation by the Sanger sequencing method for nearly all study strains because the 150 bp long Illumina sequence reads were not always sufficient for the correct assembly of this genomic region. As a result, we observed some structural variations in this region, whose lengths ranged between 12 bp to 180 bp. The repeat regions were mainly composed of five sequence motif units (designated here as ‘a’ to ‘e’). The most numerous repeats were the iterations of motif ‘a’ which was always followed downstream by motif ‘b’, whereas motifs ‘c’, ‘d’ and ‘e’ were typically positioned after ‘b’. Depending on the isolate, the number of motifs ‘a’ in this genomic region varied between one and six.

**Figure 1:**
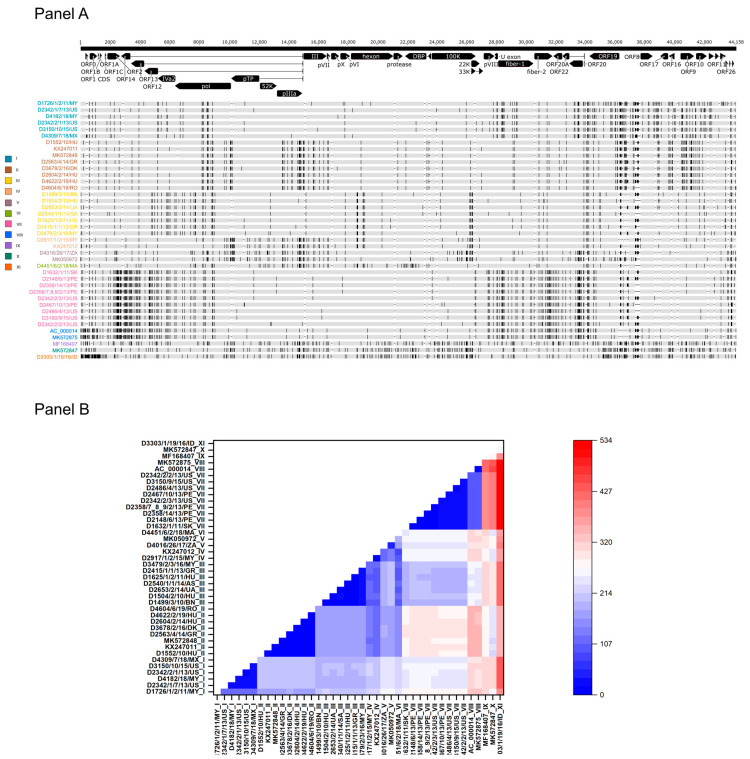
Sequence diversity within the FAdV-1 genomes. (**Panel A**) Genome types of FAdV-1 strains based on the distribution of polymorphic sites. (**Panel B**) Heat-map showing the intergenomic nucleotide differences w/o the structurally diverse tandem-repeat region.

When analyzing the genetic relationship between study strains and reference strains, low sequence diversity was observed (maximum sequence divergence between any genomes, 1.2%). In other words, when the tandem-repeat region between ORF19 and ORF8 was omitted, the number of sites where single-nucleotide variation (SNV) was recorded fell between zero and 534 nt along the entire genomes (Figure 1). Complete sequence identity was found between two strains from Peru isolated in 2013, between a strain from Hungary isolated in 2014 and a strain from Poland isolated in 2011, as well as between a Hungarian strain and a Romanian strain from 2019. The most extensive divergence was seen when a strain from Indonesia (D3303/1/19/16/ID) was compared to any other isolates (range of sequence difference, 262 to 534 nt).

When inspecting the consensus genome sequence alignment visually, we observed a peculiar pattern of the distribution of polymorphic sites (i.e., SNVs). At least 11 individual SNV patterns were observed (Figure 1). Among these, four unique patterns were identified, three in Asian countries and one in Africa. The pattern seen in the prototype FAdV-1 strain, CELO (represented by two independent GenBank records, AC_000014 and MK572875), was not found in any recent field isolates. The number of substitutions was <120 nt among strains within a particular SNV pattern; in this study, strains with a shared SNV pattern are referred to as FAdV-1 genome type (GT). Descendants of some FAdV-1 GTs were found at large geographic distances. For example, GT II strains were isolated from at least four European countries, GT I strains were detected in the Americas and Asia, whereas GT III and GT VII, respectively, were isolated from countries within Europe and Asia and within the Americas and Europe.

**Figure 2:**
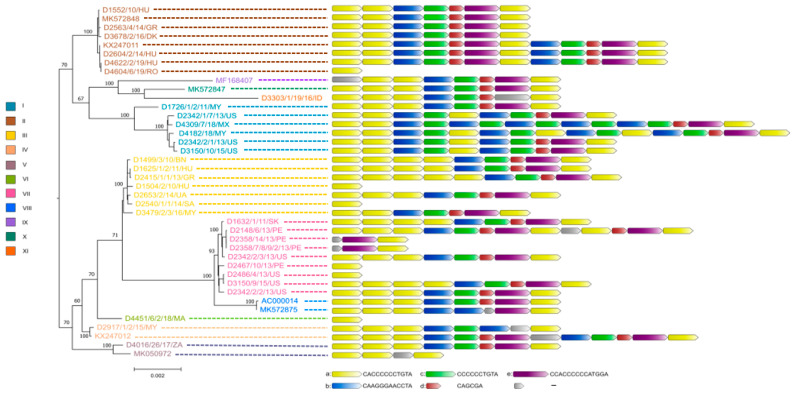
Genetic relationship between FAdV-1 strains. Whole genome-based phylogeny is shown on the left, while the structure of tandem-repeat region in different strains is shown on the right.

**Figure 3:**
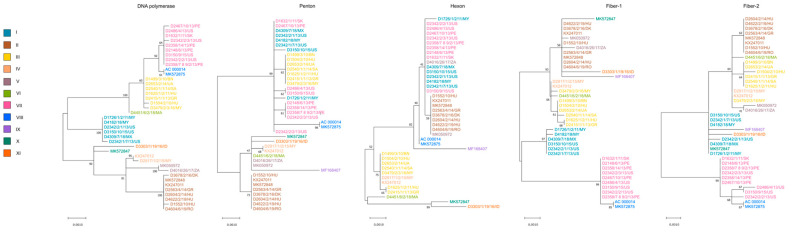
Gene-wise phylogeny using selected genes. Strains are highlighted based on their genome types.

Not surprisingly, the whole-genome-based phylogenetic tree correlated well with the SNV pattern. However, when gene-wise analyses were carried out, the location of strains in the respective trees showed a wide variation; the only exception was the tree generated from the adenoviral pol gene, which showed nearly identical topology with the whole-genome-based tree (see Figure 2 and Figure 3). In Figure 3, we highlighted the phylogeny of five marker genes, which clearly show the lack of conservation in strain clustering. The findings from gene-wise phylogenies raised the possibility that recombination may play a role in the evolution of FAdV-1 genomes.

**Figure 4:**
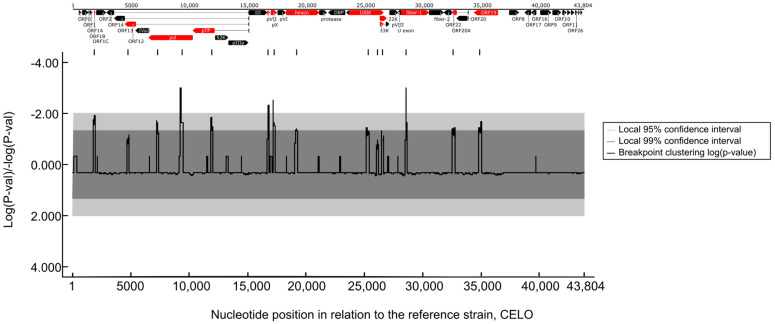
Recombination hotspots along the FAdV-1 genomes. Only the statistically significant recombination sites are shown along the position of the FAdV-1 genome. Genes where recombination events occur most likely are highlighted (red).

To confirm this, we performed recombination analyses and provided evidence for past recombination events within FAdV-1 strains. These events were identified in several genomic regions and further analyses spotted the most probable recombination breakpoint sites (Figure 4). The breakpoint sites were scattered along the entire length of the genomic DNA and affected roughly one-third of genes.

### 3.2. Genetic Features and Pathology

An attempt to connect the pathology phenotypes to virus genetic markers resulted in some intriguing observation, although we need to emphasize that the low number of analyzed strains, the incomplete dataset as well as the sampling bias resulting from the geographic representation of genetic variants may distort the final picture we delineate in this study. When analyzing pathologies from the perspective of genome-type specificities, GT II strains were more commonly isolated from gizzard erosion cases than from cases with other pathologies (gizzard erosion, 87.5%; others, 12.5%). Other pathologies mainly included IBV or IBDV co-infections with their respective clinical and pathological features; additionally, we merged the few strains originating from asymptomatic animals in this category. An inverse association between pathologies and GTs was seen for GT III (gizzard erosion, 28.5%, others, 71.5%) and GT VII (gizzard erosion, zero; others, 100%) isolates. Gene-wise phylogenies partly echoed these findings. A handful of strains systematically formed a separate branch and this branch included predominantly GT VII FAdV-1 strains (as seen in Fiber-1 and Fiber-2 trees in Figure 3) which occasionally co-clustered with GT V and/or GT VIII strains. The genes where GT VII (and facultatively, GT V and GT VIII) strains formed separate branches and these branches were supported statistically by high bootstrap value were as follows: ORF1B, ORF1C, ORF2, ORF14, ORF13, ORF12, pol, pVIII, both fiber genes, ORF20, ORF20A and ORF22. Of note, a linkage between the pathology and the structure of the tandem-repeat region located between ORF19 and ORF8 was not seen.

## 4. Discussion

In this study, we sequenced the genomes of FAdV-1 strains from a global collection. Although the relatively narrow window of detection dates and the geographic representation of isolates limited the epidemiologic evaluations, we uncovered some intriguing evolutionary features of FAdV-1 genomes, not reported earlier.

Analyzing the 40 available viral genomes and using the genome-wide distribution of polymorphic sites, we classified the reference and study FAdV-1 strains into 11 genome types. Our analyses indicated that different genome types co-circulate in parts of the world (e.g., GT II and GT III in Europe, GT I and GT VII in the Americas). Furthermore, some GTs were found in different continents (e.g., GT I in the Americas and Asia, GT III in Europe and Asia). It is possible that commercial trading of the avian hosts contributed to the spread of individual genome types but, without adequate background information in our database, we cannot confirm this hypothesis.

The tree topology seen in the viral DNA polymerase corresponded to the topology derived from the whole genomes, but no other genes (including the main neutralization antigen-coding genes) resulted in similar branching patterns of strains. Although most epidemiology studies use the hexon gene to study local strain diversity, which may be a suitable marker of strain diversity, our data suggest that the epidemiologic spread of strains might be more reliably estimated from the analysis of whole genomes or the full-length DNA polymerase genes. This illustrates that genome-wide recombination events with multiple breaking points may complicate the epidemiologic evaluation of FAdV-1 outbreaks when other genomic regions (e.g., hexon, fiber) are used as molecular epidemiology markers [17,18,19].

Both homologous and heterologous recombination have been published previously in aviadenoviruses [5,20,21]; however, the few available genome sequences seem to have prevented the recognition of such events occurring in FAdV-1 strains. In this study, we presented evidence for multiple recombination events that have occurred in the past and identified the major recombination breakpoints where genomic information exchange is most likely to occur between parental strains. Recombination in FAdV species characterized by greater genetic diversity may introduce new serological and pathological phenotypes [5]. However, the advantageous effects of recombination on viral fitness, if any, in FAdV-1 are currently unclear. If we compare the genetic distance between the genomes of the FAdV-1 strains, only limited sequence divergence (up to 1.2%) is seen, although it is also clear that even small changes in genome sequences can have a big impact on pathogenesis and virus fitness if it is in a critical position. Typically, aviadenoviruses classified into other FAdV species share greater genome sequence heterogeneities, partly due to extensive variation in the immune epitopes [5]. As reported recently, FAdV-B, another single-serotype FAdV species shows considerable genome-wide diversity without evidence of serological diversification [20,22]. An analogous immune epitope analysis of FAdV-A strains with hexon and fiber proteins that evolved through recombination appears to be relevant from the perspective of disease control and prevention once commercial vaccines become available.

The tandem-repeat sequences constitute enigmatic regions of the genome. In other FAdV species, tandem-repeat regions were found to be either dispensable for viral replication or to significantly affect the virulence [23,24]. We showed here that a set of 6- to 14-nt long sequence stretches reiterate along a ~200 bp (or shorter) region of the FAdV-1 genome located between ORF19 and ORF8. Our data indicate that the structure of these repeat regions correlates neither with the GT-specificities nor with the phylogenetic relationship between FAdV-1 strains, because intra-GT structure heterogeneities and inter-GT similarities were identified alike. The significance of this structural heterogeneity in FAdV-1 remains to be determined; however, this feature could not be linked directly to clinical manifestation/pathological alterations in our data set.

Indeed, due to the lack of sufficient background data, we could not consistently link the presence of gizzard erosion or other putative FAdV-1-associated pathologies to the genetic markers reported in this study. However, isolates belonging to GT I, II, III and IV were detected both in animals showing the typical pathologies of FAdV-1 infection as well as in animals that were diagnosed with other (i.e., non-specific) diseases. Moreover, GT II strains were more often isolated from gizzard erosion cases than other GTs. Gene-wise phylogenies led to the same conclusions. One intriguing observation was that GT VII (and occasionally GT V and GT VIII) strains were isolated from asymptomatic animals or from animals with non-specific disease, but never from gizzard erosion. In cases when non-specific pathologies were observed in GT VII strains, IBV or IBDV co-infections were diagnosed without typical FAdV-1-associated disease. At present, the role, if any, of GT VII strains in the observed pathologies is not fully understood. These strains systematically formed a common branch in at least one-fourth of gene phylogenies. Some of the implicated genes were linked to virulence in other FAdV species [24,25,26,27]. It would be interesting to study the possible role of these genes in the pathogenesis of FAdV-1-associated infections. The identification of avirulent FAdV-1 GTs could be the first step to develop novel, naturally attenuated homologous orally administered vaccines against FAdV-1-associated gizzard erosion that may act directly on the mucosal surface of the gizzard. For example, infection with the avirulent prototype FAdV-1 strains, CELO, protected chickens from gizzard erosion challenged with a pathogenic FAdV-1 strain [28].

## 5. Conclusions

The present study provides baseline information concerning FAdV-1 diversity and opens new possibilities to explore the significance of various evolutionary mechanisms in the pathology and epidemiology of these aviadenoviruses.

## Data Availability

Sequence data were deposited in GenBank under the accession numbers OP985603 to OP985634.

## Supplementary Materials

The following supporting information can be downloaded at: https://www.mdpi.com/article/10.3390/ani13182819/s1, Table S1: Background information of FAdV-1 isolates; Table S2: NGS output and some genomic features.
